# Comparison of Tau and Amyloid-β Targeted Immunotherapy Nanoparticles for Alzheimer’s Disease

**DOI:** 10.3390/biom12071001

**Published:** 2022-07-18

**Authors:** Yara Mashal, Hosam Abdelhady, Arun K. Iyer

**Affiliations:** 1 Department of Pharmaceutical Sciences, Eugene Applebaum College of Pharmacy and Health Sciences, Wayne State University, Detroit, MI 48201, USA; ymashal23@gmail.com; 2 International Academy East, Troy, MI 48085, USA; 3 Department of Physiology & Pharmacology, College of Osteopathic Medicine, Sam Houston State University, Conroe, TX 77304, USA

**Keywords:** Alzheimer’s disease (AD), nanoparticles (NPs), blood–brain barrier (BBB), drug delivery, targeted medicine, neurodegeneration

## Abstract

Alzheimer’s disease (AD) is a rapidly growing global concern associated with the accumulation of amyloid-β plaques and intracellular neurofibrillary tangles in the brain combined with a high acetylcholinesterase activity. AD diagnosis is usually made too late, when patients have an extensive neuronal death, and brain damage is irreversible. Several therapeutic targets have been defined mainly related to two hypotheses of AD: the tau hypothesis and the amyloid-β hypothesis. Here, we intend to investigate and to compare different therapeutic approaches for AD, mainly based on nanoparticles (NPs) targeted at the brain and at the pathological hallmarks of the disease. We analyzed preclinical trials that have successfully improved drug bioavailability in the brain by using targeted nanocarriers towards either tau, amyloid-β, or both. We then compared these trials to find out which protein is more efficient in therapeutic targeting. We found that the search for a cure was mostly based on the amyloid-β hypothesis, with Aβ dysplasia emerging as the most confirmed and convincing therapeutic target. Targeted NPs have proven useful to enhance both the bioavailability and the performance of therapies against AD in animal models. A better understanding of AD mechanisms will help the successful application of targeted NPs for combined therapies.

## 1. Introduction

Alzheimer’s disease (AD) is a chronic neurodegenerative disorder associated with the accumulation of amyloid-beta and intracellular neurofibrillary tangles in the brain, specifically the hippocampus. It is the most common form of dementia, constituting 60–80% of all forms. It is estimated that 5.7 million people in the U.S. have AD of whom 5.5 million are aged 65 years or older.

This makes Alzheimer’s disease the sixth leading cause of death in the U.S. Moreover, it is estimated that the number of patients over 65 years old may rise to 13.8 million by 2050 in the U.S., if the treatment of AD is not improved [1]. At first, Alzheimer’s disease takes its effect in parts of the brain involved in memory, including the entorhinal cortex and the hippocampus. It later presents in areas of the cerebral cortex, which are responsible for language, reasoning, and social behavior. Once AD reaches all lobes and areas of the brain, it starts effecting the parts of the brain that are responsible for the involuntary functions of the body. The brain starts forgetting how to breathe and pump blood and the patient becomes prone to infections as parts that control immune responses become affected.

Consequentially, a patient with Alzheimer’s gradually loses his or her ability to live and function independently over time. Ultimately, the disease is fatal [2].

### 1.1. Loss of Neuronal Connections & Cell Death in AD

**Figure 1:**
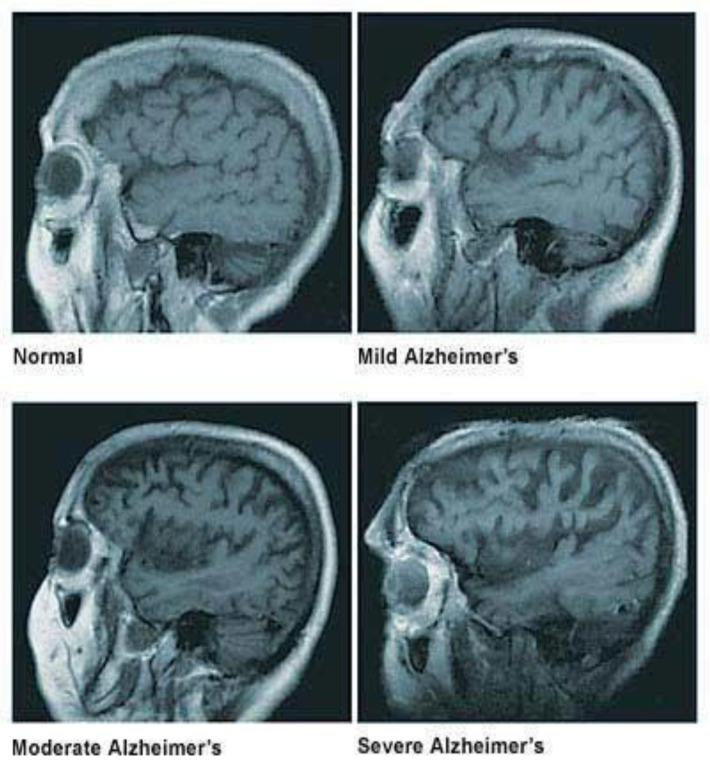
The widening grooves and fissures of the cerebral cortex indicate progressively severe brain atrophy and loss of brain mass. Adapted with permission from [3].

A healthy human brain contains tens of billions of neurons, approximately 86 billion, which are specialized cells that process and transmit information through electrical and chemical signals. These neurons send messages between different parts of the brain and from the brain to the muscles and the organs of the body [2]. Most neurons have three primary parts: a cell body, multiple dendrites, and an axon. The cell body is the neuron’s core, where it carries genetic information, maintains the neuron’s structure, and provides energy to drive activities. Dendrites are fibrous roots that branch out from the cell body, where they receive and process signals from the axons of other neurons. Lastly, the axon is a long-tailed structure which joins the cell body at a specialized junction called the axon hillock. Normally the brain shrinks to some degree in healthy aging but does not lose neurons in large numbers. On the other hand, in Alzheimer’s disease, neurons stop functioning, lose connections with other neurons, and die. It disrupts processes vital to neurons and their networks, including communication, metabolism, and repair [2]. In AD, as neurons die throughout the brain, many brain regions begin to shrink, which results in structural changes in the way the brain looks after it is affected by AD, as seen in Figure 1. By the final stages of Alzheimer’s, this process, known as brain atrophy, is widespread, causing significant loss of brain volume [3].

### 1.2. Causes & Diagnosis of AD

The exact causes of Alzheimer’s disease are not yet fully understood. The onset of the disease depends on several elements, including inheritance, sex, aging, and environmental and social factors, as well as various pathologies (i.e., atherosclerosis, hypertension, hypercholesterolemia, type II diabetes, stroke, transient ischemic attack, and brain trauma) [4]. Additionally, it has been reported that epigenetic mechanisms, such as DNA methylation or histone acetylation/deacetylation, influence memory processes and may thus contribute to AD. The first clinical manifestations of the disease are shown to be short-term memory loss, cognitive impairment, psychiatric symptoms, behavior disturbances, followed by dementia, physical impairment, long-term memory loss, and premature death.

### 1.3. Description of Tau Protein and Its Mechanisms

The tau protein hypothesis states that Alzheimer’s progression and development is also due to the aggregation of a protein known as tau. While tau is an intrinsically disordered protein and there is no specific folding related to its function, post-translational modifications (PTMs) of tau are the most responsible for abnormal or non-existent binding to microtubules, leading to tau’s aggregation; the amyloid precursor protein is structurally well-defined, and misregulation of secretases is what cause the appearance of amyloid-β peptides and consequent oligomers and fibrils.

Abnormal accumulations of tau are called neurofibrillary tangles, that collect inside neurons. The tau proteins are a group of six highly soluble protein isoforms produced by alternative splicing from the gene MAPT. In a non-AD brain, tau binds to and stabilizes microtubules. Microtubules are structures that support neurons internally, which help guide nutrients and molecules from the cell to the axon and dendrites [4,5]. Whenever these microtubules form, they extend in length, but they can also contract in which case the microtubule will essentially fall apart. The process by which microtubules fall apart is known as a microtubule catastrophe. In a non-AD brain, tau in its phosphorylated state will stabilize the microtubule in whatever length that is and prevent a microtubule catastrophe [4]. Due to this, tau should not be hyperphosphorylated, meaning it should not be excessively phosphorylated. This hyperphosphorylation of tau is what ultimately leads to the initial stimulus that leads to the failure of these microtubules and therefore AD. In the hyperphosphorylated form, net catastrophe is reached as well as failure of normal functions within the cell including cell division and trafficking, which are highly important for a normal physiological function. Not only this, but tau can also have oligomers, leading to what is known as tau oligomerization [6].

**Figure 2:**
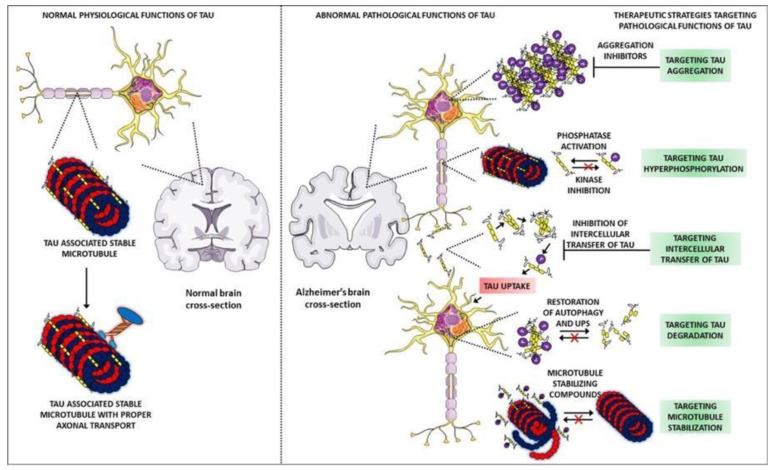
Comparison of normal and abnormal physiological functions of tau protein. Adapted from [12].

These oligomers will form a plaque-like structure, although the term ‘plaque’ is normally reserved for the Aβ hypothesis. Moreover, large aggregates form in the cytosol and in the context of tau, these have historically been called neurofibrillary tangles, as mentioned before [6]. These large aggregates of tau can exist on the intracellular side of the cell in which case they induce apoptosis. Aggregates can also be exported out of the cell, where they will themselves induce inflammation, mainly by their effect on microglial cells (glial cells of the brain and central nervous system) [7]. Whenever neurofibrillary tangles activate microglia, those microglia will release pro-inflammatory mediators, such as interleukin-1 beta (IL-1β), which are known to propagate this inflammation even further and therefore hyperactivate the kinases which lead to an increased hyperphosphorylated tau [7]. Figure 2 proposes that in terms of its function in AD, the tau protein is going to be a positive feedback cycle. In addition, more inflammation can also trigger the kinases, that is, inflammation independent of these neurofibrillary tangles [5]. There are also literature reports that indicate, other processes, such as acylation and glycosylation, causes tau malfunction [8,9,10,11]. In conclusion, although the mechanism itself is not completely understood, there is evidence that the tau protein does play a role in the neurodegeneration seen in AD.

### 1.4. Structure of Amyloid-β Peptide

**Figure 3:**
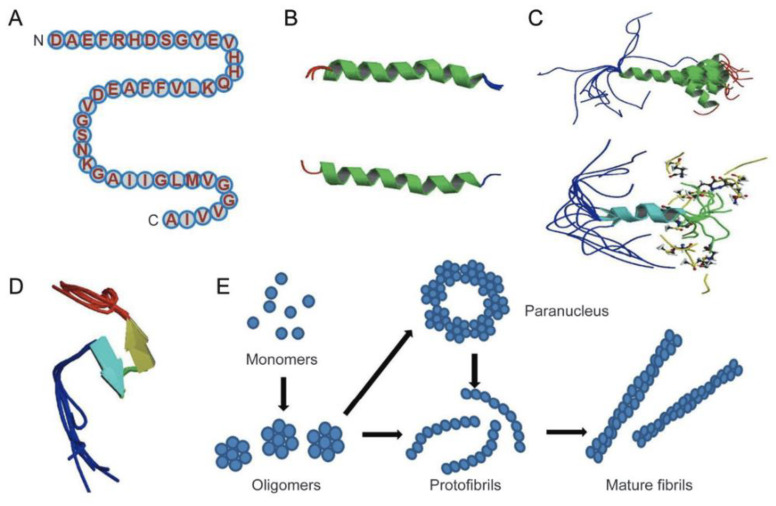
Structure of Amyloid-β monomer, oligomers, protofibrils, and fibrils. (**A**) The primary amino acid sequence of the Aβ isoform. (**B**) The structure of amyloid beta peptide in which an alpha-helical structure is apparent. (**C**) Solution structure of amyloid beta in which two-thirds of the peptide form an alpha-helix conformation. (**D**) Amyloid beta peptide forming a collapsed coil structure entailing a series of loops, strands, and turns with no alpha-helical or beta-sheet structure. (**E**) The proposed pathway of converting amyloid beta monomers to higher order oligomers, protofibrils, and fibrils. Adapted from [13].

The Amyloid-β peptides are cleaved from a much larger membrane glycoprotein precursor called APP [13]. The structures of Aβ monomer, fibril, and oligomers are shown in Figure 3 [13]. Specifically, the structure of amyloid-β peptide is shown in Figure 3B, which is majorly in α-helical structure, and it can be converted to a β-sheet in membrane-like media. Figure 3E shows the proposed pathway for the conversion of Aβ monomer to the more ordered oligomers and fibrils. In essence, the Aβ monomers can form assemblies of higher order, which can range from oligomers (of low molecular weights such as dimers to pentamers) to oligomers (of mid-range molecular weights) that could range from hexamers to protofibrils and fibrils.

### 1.5. Description of Amyloid-β Protein and Its Mechanisms

The hypothesis that endorses tau’s neurofibrillary tangles (NFTs) as the lead cause of AD states that tau hyperphosphorylation is what impairs the binding of tau-microtubules, leaving tau prone to aggregation. This hypothesis states that increased amyloid aggregation causes AD by triggering toxic events leading to progressive neurodegeneration [14,15,16]. The amyloidogenic pathway, the pathway that produces plaques of beta amyloid, is the pathway that will lead to the progression of AD. The amyloid precursor protein (APP), a protein normally existing in the plasma membrane of cells, can be cleaved by several different enzymes known as secretases [17]. There is an alpha secretase (α-secretase), beta secretase (β-secretase), and a gamma secretase (γ- secretase). As beta and alpha secretase cleave APP, they cleave toward the right side, resulting in a protein identified as P3, which is not known to be involved in AD. This is considered as the non-Alzheimer’s disease producing pathway.

**Figure 4:**
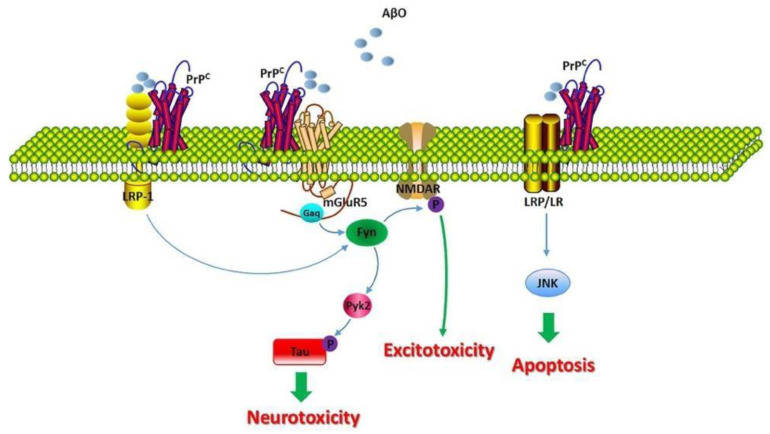
Molecular consequences of the PrPC/Aβ interaction in AD. Adapted from [19].

However, for an unknown reason, it has been determined that when alpha and gamma secretase cleave APP, they cleave in divergent ways as α-secretase and γ-secretase cleave at different locations [18]. In contrast, when beta and gamma secretase cleave APP, they cleave toward the left side, yielding two major proteins known as the amyloid intracellular domain (AICD) and amyloid-β. While AICD has no known function as yet associated with AD, amyloid-β can misfold and form Aβ oligomers that assemble and aggregate into Aβ fibrils [19]. These fibrils disrupt calcium (Ca^2+^) homeostasis and induce neuronal destruction, and ultimately the apoptosis of that neuron. The mechanism by which calcium disturbances are caused is not yet fully understood, however, Figure 4 depicts how they may interact. As shown, the healthy form of the protein, PrPC, is embedded within the membrane. The expression of PrPC is important for Aβ-induced neurotoxicity, as evidenced by the loss of long-term potentiation (LTP) and memory impairment [20,21]. Balducci, C. et al., suggests that PrPC deficiency grants resistance to the synaptic toxicity of oligomeric Aβ in mice and in vitro in hippocampal slice cultures [18].

However, Aβ fibrils can interact with PrPC, although it has been thought to interact with caveolin-1 (CAV1), a transmembrane and intracellular protein encoded by the CAV1 gene. CAV1 can thus activate p59fyn, which can then phosphorylate and activate the Nmethyl-D-aspartate (NMDA) receptor [22,23]. This is one of the mechanisms by which it has been suggested that Aβ could induce a disruption of Ca^2+^ homeostasis, considering that when a glutamate binds to the NMDA receptor, it will then induce Ca^+2^ influx into the cell [21,22]. When there is an excessive amount of Ca^2+^ influx into the cell, cytotoxicity and eventually mitochondrial dysfunction are reached, which subsequently leads to apoptosis due to the leakage of cytochrome-c or cyt-c into the cytoplasm (a direct inducer of apoptosis). Additionally, p59fyn can also phosphorylate tau (Tau pY18), resulting in an additional kinase independent of the tau protein kinase 1 (GSK3) [23]. This suggests that there is an overlap between the two hypotheses.

Moreover, Aβ can also act as an inflammatory stimulator of microglial cells that can stimulate the microglial cells to release more IL-1β, inducing additional inflammation for further activation of these kinases, which again leads to increased tau phosphorylation [24]. This suggests that development and progression may be a combination of both pathways, considering how the two proteins overlap. Ultimately, no matter which pathway is considered, it is the death of the neurons, particularly in the cerebral cortex, that leads to the deterioration and the neurodegeneration observed in AD and ultimately the death of the patient. In conclusion, amyloid-β is a part of one of the two main hypotheses for the development and the progression of AD.

### 1.6. Effects of Acetylcholinergic Neurons

Alpha-7 nicotinic acetylcholine receptors (α7nAchR), also known as α7 nicotinic receptors, are significantly reduced in AD patients, resulting in cholinergic neurons apoptosis and a reduction in the level of acetylcholine in the brain [19,25]. The enzyme in the synapses of neurons, acetylcholinesterase, naturally destroys α7nAchR [25]. If the acetylcholine producing neurons are dying in AD, then the brain would naturally want to preserve acetylcholine. Due to this, one of the treatments of AD is to administer acetylcholinesterase inhibitors [25]. By inhibiting the enzyme acetylcholinesterase, ACh degradation is significantly reduced, and what is left is preserved. This has only been shown to marginally decelerate the progression of AD, but it is by no means a cure.

### 1.7. Targeting in AD

Treatment plans targeting AD consist of drug design that include small molecules that are easy to incapsulate in NPs and easy to handle, as well as antibodies for central nervous system targeting. Amit, T. et al. found success in novel compounds that are specifically and rationally designed to cross the BBB and target multiple mechanisms underlying the specific neurodegenerative processes of AD [26].

On the other hand, non-pharmacological therapies (NPTs) include memory training, mental and social stimulation, as well as diet and exercise. In a study by Olazarán, J. et al., they found that NPTs emerge as a useful, versatile, and potentially cost-effective approach to improve outcomes and quality of life in AD and related disorders for both the person with dementia and the caregiver [27].

## 2. Nanotechnology for AD

**Figure 5:**
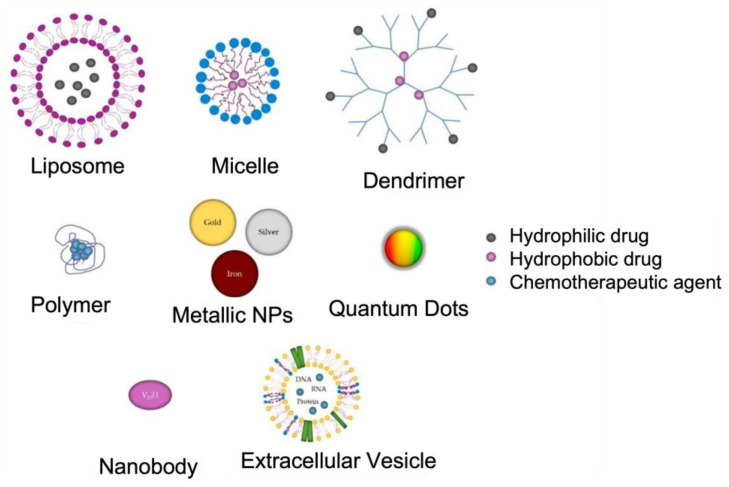
Representation of the structures of different NPs: organic (liposomes, micelles, dendrimers, and polymers), metallic (gold, silver, and iron NPs, and quantum dots), and biological (nanobodies and extracellular vesicles–exosomes). Adapted from [32].

Nanotechnology has revolutionized the approaches used to manage AD. Recently, nanotechnological advancements have the potential to offer effective diagnostic and therapeutic options. Targeted drug delivery using nanoparticles (NPs) in the range of 1–150 nm diameters can effectively cross the BBB with minimal side effects [28]. Moreover, biocompatible nanomaterials, such as those of polymeric, lipidic, and metallic origin with increased magnetic and optical properties, can act as excellent agents for early diagnosis [29]. Figure 5 depicts the main types of NPs used in the treatment of Alzheimer’s disease. The application of NPs is critical in medicine because most of the biochemical and biological mechanisms in the human body occur at the nanosize [30,31]. In addition, depending on the properties of the material, the NPs themselves can be the therapeutic or diagnostic agent (mostly magnetic and plasmonic NPs). In this review, we will examine and review the application of different types of NPs for AD targeted therapy.

In the next few sections, we will analyze some of the preclinical studies that have successfully improved drug bioavailability in the brain, using nanovectors and different targeting strategies. With that said, contrasting neuronal transmission dysfunctions cannot stop or reverse the development of AD, although they can improve the cognitive abilities of patients [29].

## 3. Polymeric NPs in the Treatment of AD

Polymeric NPs contain both a hydrophilic shell and a hydrophobic core, and they have immense potential as drug carriers as they are stable, easy-to-prepare, and readily conjugated with other molecules or drugs. Various loading methods exist for polymeric NPs, and they include loading into polymer micelles, nanoshells, and nanocapsules. The micelle’s outer layer increases its duration in the body, while the opposing interior allows for the encapsulation of water-insoluble drugs [33]. These drugs are released via the biodegradable properties of micelles, which leads to drug delivery through diffusion or decomposition over time, allowing for better penetration of the BBB [33].

Polymeric NPs with PEG and antibodies have been successfully designed and tested in transgenic AD mice. A recent study reveals that exposure of PEGylated NPs can lead to the correction of memory defect and a significant reduction in Aβ soluble peptides [28]. Thus, the designed formulation can be used to cure AD illness, according to Carradori et al. [34]. In addition, researchers are directed to a review on polyglutamic acid (PGA) that is useful for drug delivery as PGA polymer has a high potential due to its unique structure and its useful properties as a nanomaterial for application, including AD therapeutics [35].

### 3.1. Polymeric Porous Nanomaterial for AD Biomarker Analysis

In one study, authors used a nanomaterial for finding AD biomarkers from human serum that would be useful for molecular biology processes related to AD pathogenesis [1]. For this purpose, a porous nanomaterial functionalized with polyethyleneimine and Ti^4+^, denoted as H–CeO_2_@PEI-Ti, was developed. This porous hollow nanomaterial integrated several enrichment activities such as immobilized metal ion affinity chromatography (IMAC) and metal oxide affinity chromatography (MOAC) that could be applied for phosphoproteomics. The experiment results in high selectivity and good enrichment due to the large surface area of the hollow nanomaterial. In serum analyses, 49 phosphopeptides corresponding to 31 phosphoproteins and 49 phosphopeptides corresponding to 32 phosphoproteins were identified in controls and AD patients, respectively. The outcome of the study revealed that the enrichment for human serum supported processes, such as copper ion binding, heparin binding, and retinoid metabolism, that could be related to AD pathogenesis [1].

### 3.2. Polymeric NPs in Tau Targeted Immunotherapy

Liu et al. reported a multifunctional nanocarrier for a therapeutic gene and peptide co-delivery that addresses the two hallmarks of AD: Aβ plaque deposition and inhibition of tau-related fibrillar formation [36]. They were able to develop the multifunctional nanocarrier by achieving therapeutic gene and peptide co-delivery to the brain based on PEGylated dendrigraft poly-l-lysines (DGLs) via systemic administration, where the dendritic structure of DGLs provides a positive charge and plenty of reaction sites for drug loading [36]. Their study was completed by using multiple-dosing treatments in transgenic AD mice. With both in vitro and in vivo experiments, the multifunctional nanocarrier proved to be an excellent drug co-delivery platform for AD. More information on this study is seen in the first row of Table 1.

Another study published in 2021 by Zhu, L. et al. also reported a multifunctional nanoinhibitor based on self-assembled polymeric micelles. Through the multivalent binding effect with the aggregating protein, their nanoinhibitor was capable of efficiently inhibiting tau protein aggregation, recognizing tau aggregates, and blocking their seeding in neural cells, thus remarkably mitigating tau-mediated cytotoxicity [37]. Their nanoinhibtor was additionally decorated with tau-binding peptide and thus the formed nanoinhibitor–tau complex after binding is more easily degraded than mature tau aggregates. Their results led them to believe that this multifunctional nanoinhibitor will promote the development of new antitau strategies for AD treatment [37]. More information on this study is seen in Table 1.

### 3.3. Polymeric NPs in Amyloid-β Targeted Immunotherapy

Zhang, C. et al. developed a dual-functional nanoparticle drug delivery system loaded with β-sheet breaker peptide H102 (TQNP/H102) [38]. Two targeting peptides, TGN and QSH, were conjugated to the surface of the NPs for BBB transport and Aβ42 targeting, respectively. The results were exemplary. The spatial learning and the memory of the AD model mice in the TQNP/H102 group were significantly improved compared to the AD control group, and they were also better than other preparations with the same dosage [38]. More information on this study is seen in Table 2.

## 4. Liposome NPs

Liposome NPs are self-assembled, amphiphilic nanovesicles composed of phospholipid bilayers or other similar amphipathic lipids. Due to their excellent biocompatibility, biodegradability, and low toxicity, and their ability to carry various types of therapeutic molecules across the blood-brain barrier (BBB) and into brain cells, they have been recently utilized for AD therapy [39,40].

Liposomes can be surface modulated using several polyether, functional proteins, as well as cell-penetrating peptides that help in drug transportation across the BBB. An example of this would be polyethylene glycol (PEG)-coated liposomes that have been reported to successfully evade the opsonization of RES. In addition, glutathione-PEGylated liposomes are also reported, by Wong et al. and Rip et al., to enhance the cellular uptake of the drug across endothelial BBB [41,42].

### 4.1. Liposome NPs in Tau Targeted Immunotherapy

In a preclinical study by Theunis, C. et al. that was published in 2013, inflammatory markers have been reported to lead to negative outcomes. In this study, they adapted the liposome-based amyloid vaccine that proved safe and efficacious, and they incorporated a synthetic phosphorylated peptide to mimic the important phospho-epitope of protein tau at residues pS396/pS404 [43]. They showed that the liposome-based vaccine produced specific antisera in wild-type mice and in tau.P301L mice. Their findings support that the liposome carrying phosphorylated peptides of tau are a considerably safe and effective treatment against tauopathies, including AD. More information on this is seen in Table 1.

In another study by Gao, C. et al. that was published in 2020 used curcumin under the synergistic effects of T807 to effectively penetrate the BBB as well as bind to hyperphosphorylated tau in nerve cells, where they inhibit multiple key pathways in tau-associated AD pathogenesis. In their study, it was observed that CUR-loaded T807/RPCNP NPs can relieve AD symptoms by reducing p-tau levels and suppressing neuronal-like cell death both in vitro and in vivo [16]. As a result, the memory impairment observed in an AD mouse model was significantly improved, following systemic administration of CUR-loaded T807/RPCNP NPs. More information on this study is seen in the fourth row of Table 1.

### 4.2. Liposome NPs in Amyloid-β Targeted Immunotherapy

Among the small molecule drugs reported for AD, curcumin, a compound derived from the dietary spice turmeric, has been the most used by research groups. Curcumin is a polyphenolic compound derived from the dietary spice turmeric. Lazar, A. N. et al. and Fan, S. et al. [44,45] have reported that curcumin-conjugated nanoliposomes may be applied in the diagnosis of AD and related targeted drug delivery, with its anti-amyloid, anti-inflammatory, and anti-oxidant properties. Fan, S. et al. designed novel, brain-targeted NPs made of poly(lactide-co-glycolide)-block-poly(ethylene glycol) (PLGA- PEG) and conjugated it with B6 peptide. These NPs were then loaded with Cur (PLGA-PEG-B6/Cur) and used for HT22 cells and APP/PS1 Al transgenic mice. They found that PLGA-PEG-B6/Cur could tremendously improve the spatial learning and the memory capability of APP/PS1 mice, compared with native Cur [45]. More information on this study is seen in Table 2.

An another study, using Methoxy-XO4, by Tanifum, E. A. et al., showed that the particles maintain binding profiles to synthetic Aβ aggregates similar to the free ligand, and selectively bind Aβ plaque deposits in brain tissue sections of an AD mouse model (APP/PSEN1 transgenic mice), with high efficiency [46]. When the NPs are administered intravenously, they cross the BBB and bind to Aβ plaques. Through the synthesis of an Aβ-targeted lipid conjugate, they incorporated it in stealth liposomal NPs and tested their ability to bind amyloid plaque deposits in an AD mouse model. More information on this study is seen in Table 2.

## 5. Metallic NPs

Metallic NPs are efficient because of their tunable size and shape, low cytotoxicity, and stable attachment to ligands and molecules. These NPs can also control the release of the drug with internal or external stimuli [47].

Concerning AD, the use of metallic NPs also falls into the category of targeted drug delivery across the BBB. Because of chemistry-based techniques in their synthesis, metallic NPs have some risks, but some of the metallic NPs like cerium, selenium, gold, and iron are found to exhibit significant anti-AD properties [28]. Those risks would include interference with homeostasis, by affecting bodily functions and forming salts in the body.

### 5.1. Metallic NPs in Tau Targeted Immunotherapy

In a remarkable study conducted by Sonawane, S. K. et al. that was published in 2019, they screened protein-capped (PC) metal NPs for their potency in inhibiting tau aggregation in vitro. While never done before, they presented a novel function of PC-Fe_3_O_4_ and PC-CdS NPs as potent tau aggregation inhibitors by fluorescence spectrometry, sodium dodecyl sulfate-polyacrylamide gel electrophoresis, and electron microscopy [48]. As a result of their findings, the NPs can take a lead as potent tau aggregation inhibitors, and they can be modified for specific drug delivery due to their very small size. Their current work has shown unprecedented strategies in designing anti-tau aggregation drugs, which furthers our understanding of biological nanostructures in AD. More information on this study is seen in the fifth row of Table 1.

Another study published in 2020 by Razzino, C. A. et al., reports a new strategy for the determination of tau involving a gold nanoparticle [49]. They also used a sandwich immunoassay and amperometric detection at disposable screen-printed carbon electrodes (SPCEs) as well as the immobilization onto electrografted *p*-aminobenzoic acid (*p*-ABA). The capture antibody (CAb) was immobilized by crosslinking with glutaraldehyde (GA) on the amino groups of the 3D-Au-PAMAM-*p*-ABA-SPCE, where tau protein was sandwiched with a secondary antibody labeled with horseradish peroxidase (HRP-DAb). Their results allowed the direct determination of the target protein in raw plasma samples and in brain tissue extracts from healthy individuals and post mortem diagnosed AD patients, while using a simple and fast protocol. More information on this study is seen in Table 1.

### 5.2. Metallic NPs in Amyloid-β Targeted Immunotherapy

In a study by Nan Gao et al. that was published in 2014, gold NPs acted as multifunctional therapeutic agents for treatment of AD. After designing a novel multifunctional Aβ inhibitor, AuNPs@POMD-pep shows synergistic effects in inhibiting Aβ aggregation, dissociating Aβ fibrils, and decreasing Aβ-mediated peroxidase activity and Aβ-induced cytotoxicity [50]. By taking advantage of Au NPs as vehicles that can cross the BBB, AuNPs@POMD-pep can thus cross the BBB and overcome the drawbacks of small-molecule anti-AD drugs. More information on this study is seen in Table 2.

In another study conducted by Prades, R. et al. that was published in 2012, results were highly relevant for the therapeutic applications of gold NPs for molecular surgery in the treatment of AD. In their study, they demonstrated that gold NPs conjugated to the peptide CLPFFD are useful to destroy the toxic aggregates of β-amyloid, similar to those found in the brains of patients with Alzheimer’s disease [51]. They further introduced the peptide sequence THRPPMWSPVWP into the gold nanoparticle–CLPFFD conjugate, as it interacted with the transferrin receptor present in the microvascular endothelial cells of the BBB, thus causing an increase in the permeability of the conjugate in brain, as shown by experiments in vitro and in vivo [51]. More information on this study is seen in the eleventh row of Table 2.

**Table 1:** NPs targeted to tau.

Type of Carrier	Composition	Size (nm)	Route of Admin.	Ligand BBB	Ligand AD Target	Drug Loaded	Function	Reference
Polymeric	DGL-PEG	95–110	I.v.	Peptide (RVG29)	Peptide (RVG29)	pshBACE1-AS, Peptide (D-Pep)	Downregulation of BACE1 level inhibition of phosphorylated- tau related fibril	[29]
Polymeric (micelles)	hydrophobic poly(ε-caprolacton), hydrophilic poly (ethylene glycol)	33	In vitro	-	Peptide (D)-TLKIVW		Tau-targeted multifunctional inhibitor	[30]
Liposome	DMPC, DMPG, CH, MLPA		S.c.	-	-	Peptide (tau-fragment)	Tau immunotherapy	[36]
Liposome	RBCm-coated	150–200	I.v.	T807	Curcumin	CUR-loaded T807/RPCNP NPs	Tau targeting	[37]
Metallic	protein-capped	50–60	In vitro	-	-	10% SDS-PAGE	Tau aggregation inhibitor	[42]
Metallic (gold)	poly(amidoamine) (PAMAM) dendrimer nanocomposite (3D-Au-PAMAM)	2–100			Antibody (CAb), antibody (HRP-DAb)	-	Direct determination of tau	[43]
Carbon-based	PX@OMCN–PEG, PX@OP@RVG	110	In vivo and in vitro	RVG peptide	RVG peptide	Protoporphyrin IX (PX)	Tau phosphorylation inhibitor	[52]

**Table 2:** NPs targeted to Amyloid-β.

Type of Carrier	Composition	Size (nm)	Route of Admin.	Ligand BBB	Ligand AD Target	Drug Loaded	Function	Reference
Liposome	DPPC, CH, DPS-curcumin.	200	I.c.v.	-	Curcumin	-	Targeting Aβ	[38,39]
Liposome	DPPC, CH, DSPE-PEG	150	I.v.	Passive targeting	Methoxy XO4	-	Ttargeting Aβ	[40]
Liposome	PC or DSPC, CH, DSPE- PEG	140–170	In vitro	Antibody (OX26Mab)	Antibody (AβMab)	-	Targeting Aβ	[46]
Liposome	SM, CH, DMPA, DSPE- PEG	130	In vitro	Antibody (RI7217)	PA	-	Targeting Aβ	[47]
Liposome	DSPC, CH, DSPE-PEG	90–120	I.v.	Antibody (OX26Mab)	Antibody (19B8MAb)	-	Targeting Aβ	[48]
Liposome	SM, CH, DMPA	100–150	I.p.	Peptide (mApoE)	PA	-	Destabilize brain Aβ aggregates and promote peptide removal	[49]
Liposome	DMPC	20–35	I.v.	Protein (ApoE3)	Protein (ApoE3)	-	Decreased amyloid deposition	[50]
Liposome	PC, DSPE- PEG	80–300	I.v.	Protein (lactoferrin)	-	Peptide (KLVFF) and curcumin	β-sheet breaker antioxidant	[51]
Polymeric	PEG-PLA	90–110	I.v.	Peptide (TGN)	D-Peptide (D-QSH)	Peptide (H102)		[31]
Metallic (gold)	Au	20–25	I.v.	Passive	Peptide (LPFFD)	POMD	Inhibition of aggregation Dissociation antioxidant	[44]
Metallic (gold)	Au	15	I.v.	Peptide (THR)	Peptide (LPFFD)	-	Inhibition of aggregation	[45]

## 6. Conclusions

Unfortunately, the search for a cure for Alzheimer’s disease has been ongoing for over 100 years. AD is still not well understood, and it could be described as a multifactorial disease.

Treatment directed to amyloid and tau protein may be individually effective, but the progression of these protein’s pathologies suggest that combination therapy may be required, especially in late stages where both are aggregate/lesion abundant.

### 6.1. Comparison between Tau and Amyloid-β

Amyloid-β peptides are fragments of the transmembrane amyloid precursor protein, whereas tau is a brain-specific, axon-enriched, microtubule-associated protein. The tau hypothesis postulates that tau tangle pathology precedes Aβ plaque formation, and that tau phosphorylation and aggregation is the main cause of neurodegeneration in AD.

Targeting either protein has its advantages and disadvantages. While targeting tau, a major advantage is that it would target a nearly neuron-specific protein and thereby likely not harm most nonneuronal brain cells or organs other than the brain. However, to make this strategy possible, the challenge of delivering antisense oligonucleotides to the brain side of the BBB must first be overcome. While selectively targeting Amyloid-β, a major advantage is the lower risk of vasogenic edema and its potential complications. However, anti-amyloid antibodies have limited brain penetration and may not reach and sustain brain concentrations to effectively remove Aβ oligomers.

As a result of our findings, the search for a cure, also from the nanotechnological point of view, has been prominently based on the amyloid-β hypothesis, which is currently under question. Aβ dyshomeostasis has emerged as the most extensively validated and compelling therapeutic target.

### 6.2. Future Outlook with Nanotechnology, Molecular Modeling and Simulation

The studies we examined on drug delivery based on the use of nanocarriers show that nanotechnology could represent an important ally of medicine. NPs have for decades been used for extending the circulation time, stability, and targeted delivery of drugs; but, in recent years, NPs have been applied to improve the permeation of drugs through the BBB, especially for larger molecules. With the high volume of research coming in support of the effective usage of NP based drug delivery in the critical environment of CNS, it is quite likely that this approach can end up providing remarkable breakthroughs in the diagnosis and the therapy of AD. In addition, the ever-developing areas of molecular modeling and simulation at the nanoscale (including molecular dynamics simulations and other computational techniques) will help to provide deeper insights of interactions, structure, and function at a molecular level that could pave the way forward to target these neuroproteins under study.

## Data Availability

Not applicable.
